# Supplementary material for: Deep neural network-estimated electrocardiographic age as a mortality predictor
Source: Nat Commun. 2021 Aug 25;12:5117. doi: 10.1038/s41467-021-25351-7 (PMC8387361; doi:10.1038/s41467-021-25351-7)

Supplementary Material

Deep neural network-estimated electrocardiographic age as a mortality predictor

Emilly M Lima, MSc[1,2 *]; Antônio H Ribeiro, PhD[3,4 *]; Gabriela MM Paixão, MD, MSc[1,2,*]; Manoel Horta Ribeiro[5]; Marcelo M Pinto Filho, MD, PhD[1,2]; Paulo R Gomes, MSc[1,2]; Derick M Oliveira, MSc[3]; Ester C Sabino, MD, PhD[6]; Bruce B Duncan, MD, PhD[7]; Luana Giatti, MD, PhD[2]; Sandhi M Barreto, MD, PhD[2]; Wagner Meira Jr, PhD[3]; Thomas B Schön, PhD[4] ; Antonio Luiz P Ribeiro, MD, PhD[1,2]

1- Telehealth Center, Hospital das Clínicas, Universidade Federal de Minas Gerais, Belo Horizonte, Brazil.

2- Faculdade de Medicina, Universidade Federal de Minas Gerais, Belo Horizonte, Brazil.

3- Departamento de Ciência da Computação. Universidade Federal de Minas Gerais, Belo Horizonte, Brazil

4- Department of Information Technology, Uppsala University, Sweden.

5- Ecole Polytechnique Fédérale de Lausanne, Lausanne, Switzerland.

6- Instituto de Medicina Tropical da Faculdade de Medicina da Universidade de São Paulo, São Paulo, Brazil.

7- Programa de Pós-Graduação em Epidemiologia and Hospital de Clínicas de Porto Alegre, Universidade Federal do Rio Grande do Sul, Porto Alegre, Brazil.

*These authors contributed equally.

Corresponding authors:

Antonio Luiz Pinho Ribeiro, Telehealth Center, Hospital das Clínicas da UFMG
Av. Alfredo Balena, 110, sala 106 Sul, Belo Horizonte - MG, 30130-100, Brazil
Tel.: +55 31 3307 9201; Mobile: +55 31 987090451. Email: antonio.ribeiro@ebserh.gov.br

Thomas Schön, Department of Information Technology Uppsala University
Box 337, SE-751 05 Uppsala, Sweden
Phone: +46 18 - 471 2594 E-mail: thomas.schon@it.uu.se

Supplementary Table 1: Baseline characteristics by ECG-age groups. Display baseline characteristics for three groups of patients: those with ECG-age more than 8 years greater than the chronological age (denoted by: ">8 years older") ; those with ECG-age within a range of 8 years from their chronological age (denoted by: "± 8 years"); and, those with ECG-age more than 8 years smaller than the chronological age (denoted by: ">8 years younger").

	CODE-15%			ELSA-Brasil			SaMi-Trop		
**Characteristics**	± 8 years (n=125706)	>8 years younger (n=39455)	>8 years older (n=53008)	± 8 years (n=7710)	>8 years younger (n=5353)	>8 years older (n=1200)	± 8 years (n=796)	>8 years younger (n=307)	>8 years older (n=528)
Sex, Male, n (%)	50821 (40)	15508 (39)	22179 (42)	3403 (44)	2536 (47)	555 (46)	259 (33)	102 (33)	189 (36)
Age, years, mean (s.d.)	50.5 (20)	63.0 (15)	42.2 (17)	49.0 (12)	55.0 (13)	48.5 (12)	62.0 (20)	64.0 (19)	54.0 (16)
Hypertension, n (%)	36418 (29)	15604 (40)	12745 (24)	2695 (35)	1902 (36)	511 (43)	288 (36)	120 (39)	185 (35)
Diabetes, n (%)	7808 (6)	3462 (9)	2450 (5)	1439 (19)	1121 (21)	280 (23)	81 (10)	38 (12)	42 (8)
Smoking, n (%)	7595 (6)	2522 (6)	3528 (7)	1065 (14)	610 (11)	207 (17)	239 (30)	92 (30)	167 (32)
Previous myocardial infarction, n (%)	885 (0.7)	358 (0.9)	310 (0.6)	132 (1.7)	95 (1.8)	31 (2.6)	40 (5.0)	7 (2.3)	29 (5.5)

Data are expressed as numbers (percentage) unless otherwise indicated

Supplementary Table 2: Medical doctors discerning the ECG-age. The table displays the results for the ECG reading experiment. Medical doctors annotated 134 ECGs in three rounds. Given two options A and B, they had to decide which one had an ECG-age more than 8 years greater than their chronological age. Doctors were given the chronological age of the two patients (which were the same), and two traces. In Stage 2, doctors were given the answer after accomplishing the task (i.e., whether their assessment was correct), this yielded no difference in Stage 3. Overall, these results suggest that ECG-age captures signals that are non-trivial for doctors to distinguish.

		Stage 1<br>n=45<br>Acc=64.4%		Stage 2<br>n=45<br>Acc=62.2%		Stage 3<br>n=44<br>Acc=45.5%		Aggregated<br>n=134	
		Correct Answer							
		A	B	A	B	A	B	A	B
Given Answer	A	19	9	12	6	8	10	41	26
	B	7	10	11	16	14	12	30	37
		chisq=2.1, p=0.15		chisq=1.96 p=0.16		chisq=0.94 p=0.76		chisq=3.0, p=0.08	

Supplementary Figure 1: Saliency maps. Illustrative example of ECGs with saliency maps. Saliency maps are displayed overlayed with the ECG signal. The size of the blue dots superimposed with the ECG trace is proportional to the partial derivative of the ECG-age prediction regarding that point of the input tracing.

A)

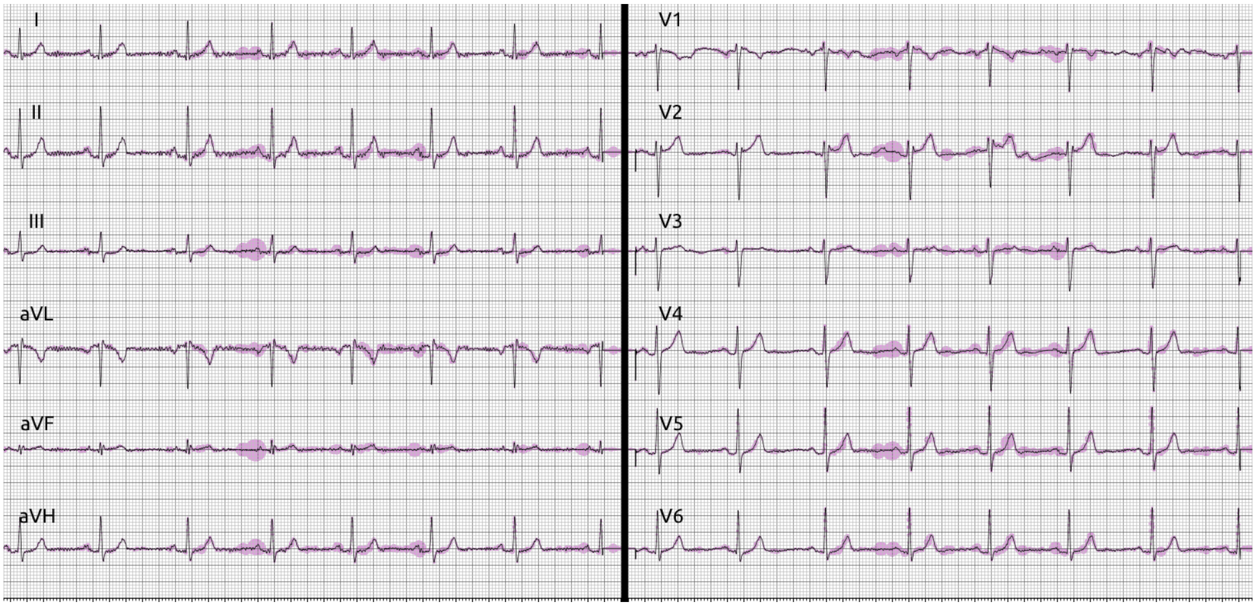

B)

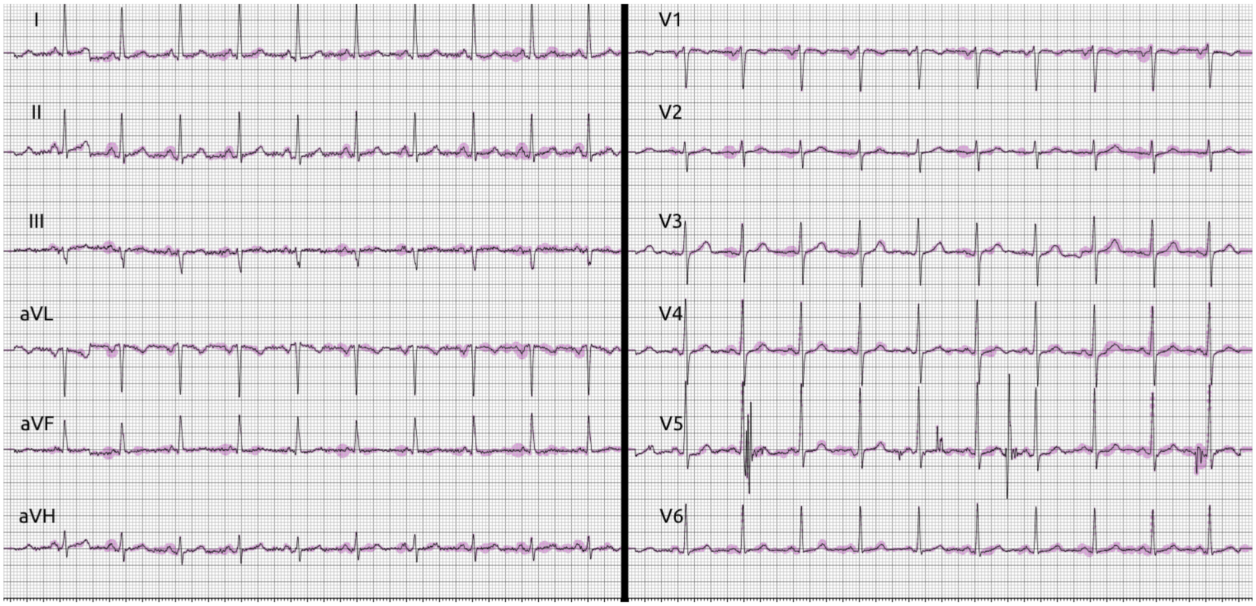

Supplementary Figure 2: Saliency maps in the frequency domain. We analyze the local sensitivity in the frequency domain and show the relative importance of each frequency component in the DNN prediction (see Methods for a precise interpretation). The analysis is performed for 100 normal ECGs randomly sampled from CODE-15%, ELSA-Brasil, and Sami-Trop with similar results. The full line is the median and the shaded region gives the interquartile range over the 100 evaluated samples.

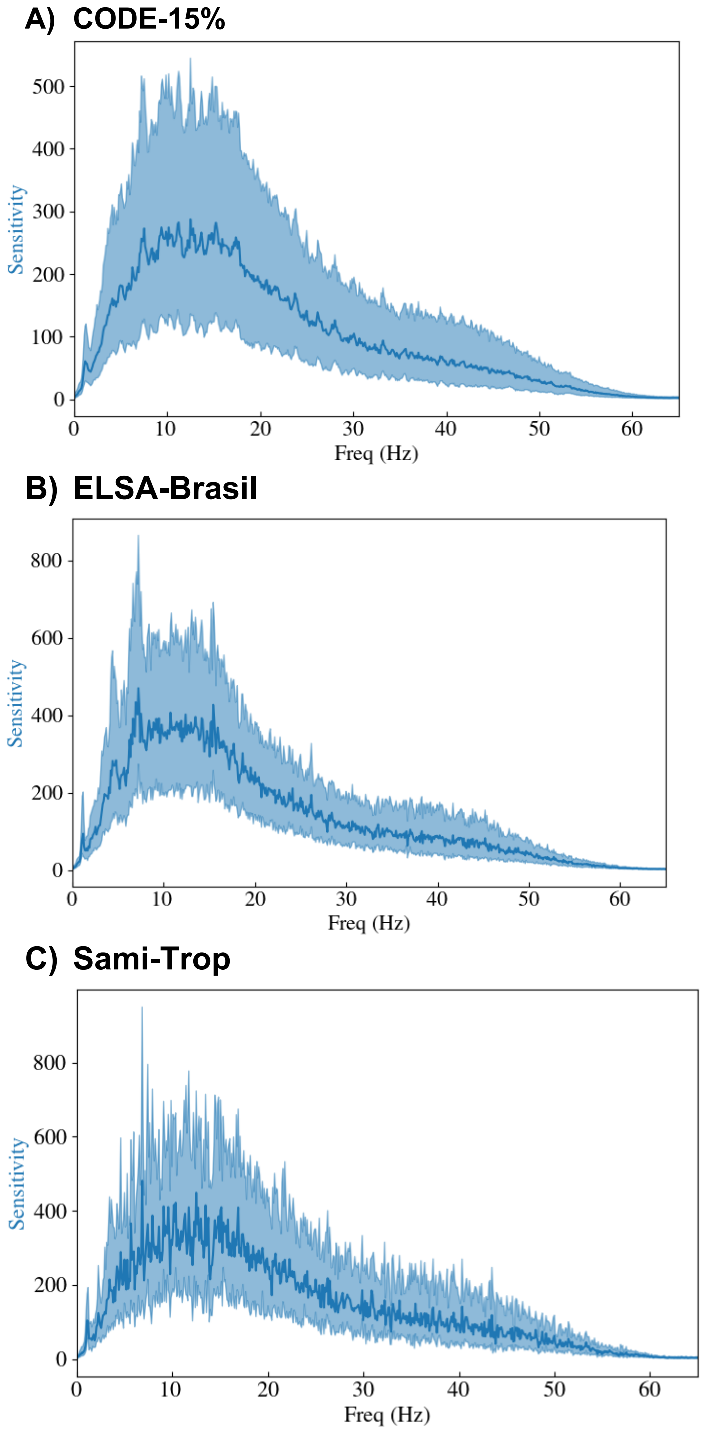

Supplementary Figure 3: Learning curve. In blue, the plot displays the mean absolute error (MAE) computed in the 5% validation set. In gray, it shows the learning rate used. The x-axis gives the epochs: each epoch a full pass through the set of training examples updating the model weights. The best model is obtained in epoch 66 with MAE=8.5097 on the validation set.

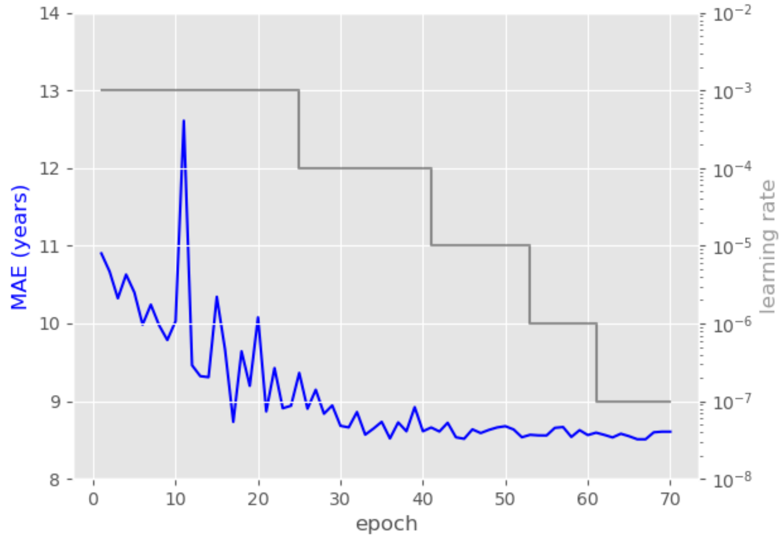

Supplementary Fig 4. Adjusted survival curve for patients' quintiles. Adjusted survival curves, hazard ratios (HR) and CIs 95% for 5 groups of the difference between ECG-age and chronological age. The patients were divided into quintiles according to quintiles of the difference of ECG-age and chronological age. The HR summarizes the Cox regression models obtained for overall mortality. The models were adjusted by age and sex.

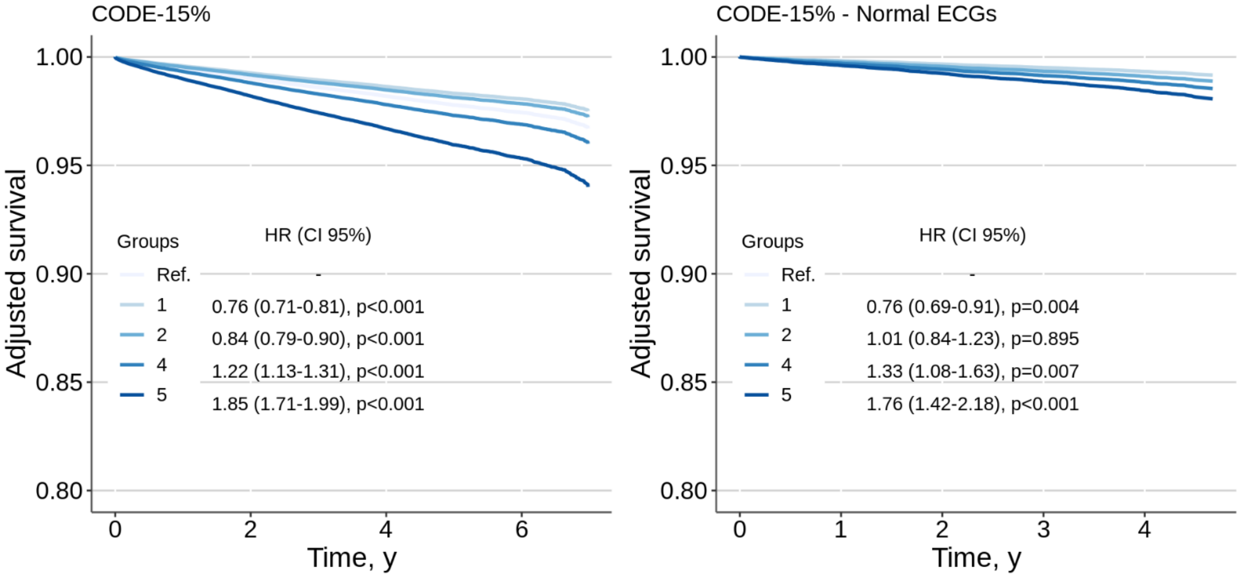

No. at risk

▬	43633	33497	16803	5853
▬	43634	33493	17200	6244
▬	43634	33507	17174	6097
▬	43634	32956	15960	5305
▬	43634	33047	16224	5394

No. at risk

▬	16810	15793	11725	7456	3219
▬	15458	14489	10717	6808	3095
▬	16052	15101	11169	7128	3140
▬	17507	16424	11997	7468	3143
▬	14852	13925	10158	6428	2744

Supplementary Fig 5: Illustrative representation of stratified sampling. We illustrate the stratified sampling used to generate the CODE-15% dataset. Each ECG exam is represented by a disk. The exams are divided into age groups with one group for each age, ranging from 16 to 85 years old. The same number of samples from each age group is then randomly picked to be assigned to the CODE-15% dataset. The same procedure is used to generate the 5% split used for validating the model.

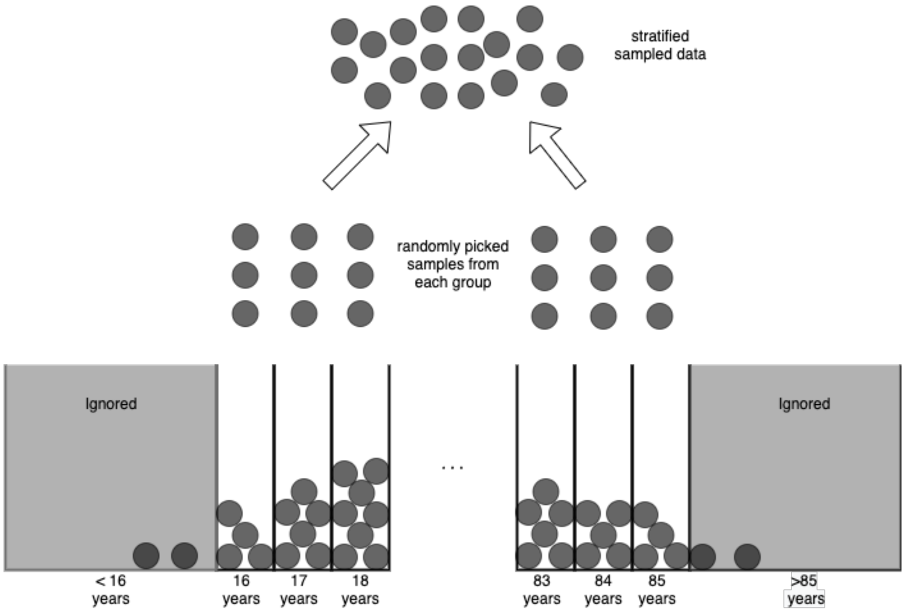

Supplement: Supplementary file 1 — Supplementary Information file [file 41467_2021_25351_MOESM1_ESM.pdf]
