# Supplementary material for: Deep neural network-estimated electrocardiographic age as a mortality predictor
Source: Nat Commun. 2021 Aug 25;12:5117. doi: 10.1038/s41467-021-25351-7 (PMC8387361; doi:10.1038/s41467-021-25351-7)
Supplement: Supplementary file 2 — Reporting Summary [file 41467_2021_25351_MOESM2_ESM.pdf]

nature research

Corresponding author(s): Antonio Luiz Pinho Ribeiro

Last updated by author(s): Jul 13, 2021

Reporting Summary

Nature Research wishes to improve the reproducibility of the work that we publish. This form provides structure for consistency and transparency in reporting. For further information on Nature Research policies, see our Editorial Policies and the Editorial Policy Checklist.

Statistics

For all statistical analyses, confirm that the following items are present in the figure legend, table legend, main text, or Methods section.

n/a	Confirmed	
☐	☒	The exact sample size (*n*) for each experimental group/condition, given as a discrete number and unit of measurement
☐	☒	A statement on whether measurements were taken from distinct samples or whether the same sample was measured repeatedly
☒	☐	The statistical test(s) used AND whether they are one- or two-sided<br>*Only common tests should be described solely by name; describe more complex techniques in the Methods section.*
☐	☒	A description of all covariates tested
☐	☒	A description of any assumptions or corrections, such as tests of normality and adjustment for multiple comparisons
☐	☒	A full description of the statistical parameters including central tendency (e.g. means) or other basic estimates (e.g. regression coefficient) AND variation (e.g. standard deviation) or associated estimates of uncertainty (e.g. confidence intervals)
☐	☒	For null hypothesis testing, the test statistic (e.g. *F*, *t*, *r*) with confidence intervals, effect sizes, degrees of freedom and *P* value noted<br>*Give P values as exact values whenever suitable.*
☒	☐	For Bayesian analysis, information on the choice of priors and Markov chain Monte Carlo settings
☒	☐	For hierarchical and complex designs, identification of the appropriate level for tests and full reporting of outcomes
☐	☒	Estimates of effect sizes (e.g. Cohen's *d*, Pearson's *r*), indicating how they were calculated

Our web collection on statistics for biologists contains articles on many of the points above.

Software and code

Policy information about availability of computer code

Data collection	Telehealth network infrastructure and software was used for collecting and storing the data. This software was developed in-house and it is not the object of this study. ECG measurements were made using the University of Glasgow (Uni-G) ECG analysis program, version 28.5, from Fri, 31 Jan 2014 (Macfarlane, P. W., Devine, B. & Clark, E. The university of glasgow (Uni-G) ECG analysis program. in Computers in Cardiology 451–454, 2005).
Data analysis	Custom software was used for for training, validating and testing our model. This custom software is openly available and was built on Python and R using standard, open-source, scientific libraries. Check code availability statement

For manuscripts utilizing custom algorithms or software that are central to the research but not yet described in published literature, software must be made available to editors and reviewers. We strongly encourage code deposition in a community repository (e.g. GitHub). See the Nature Research guidelines for submitting code & software for further information.

Data

Policy information about availability of data

All manuscripts must include a data availability statement. This statement should provide the following information, where applicable:

- Accession codes, unique identifiers, or web links for publicly available datasets
- A list of figures that have associated raw data
- A description of any restrictions on data availability

Upon publication, some of the cohorts used in the model evaluation will be made available. Information about mortality, age, sex, the ECG tracings and the flag indicating whether the ECG tracing is normal will be made available with no restriction for the Sami-Trop cohort and for the CODE-15% cohorts. The DNN model parameters that give the results presented in this paper will also be made available without restrictions. This should allow the reader to partially reproduce the results presented in the paper. Restrictions apply to additional clinical information on these two cohorts, to the full CODE cohort and to the ELSA-Brasil cohort, for

which requests will be considered on an individual basis by the Telehealth Network of Minas Gerais and by ELSA-Brasil Steering Committee. Any data use will be restricted to non-commercial research purposes, and the data will only be made available on the execution of appropriate data use agreements.

Field-specific reporting

Please select the one below that is the best fit for your research. If you are not sure, read the appropriate sections before making your selection.

- [x] Life sciences
- [ ] Behavioural & social sciences
- [ ] Ecological, evolutionary & environmental sciences

For a reference copy of the document with all sections, see nature.com/documents/nr-reporting-summary-flat.pdf

Life sciences study design

All studies must disclose on these points even when the disclosure is negative.

Sample size	The CODE cohort (n=1,558,415 patients) was used for training and evaluating the model. The external cohorts ELSA-Brasil (n=14,236) and SaMi-Trop (n=1,631) were used to validate the results. Sample size calculation was not performed considering the large amount of data available and that all these studies had calculated their sample size for less frequent outcomes.
Data exclusions	In the CODE cohort, patients under 16 years old were excluded, considering that the reference values and criteria for diagnosis in children and adolescents are different from those used in adults. ECG tracings with technical problems that impaired the medical report were also excluded.<br><br>In the ELSA-Brasil cohort, exclusion criteria were severe cognitive or communication impairment, intention to quit work at the institution in the near future for reasons not related to retirement, and, if retired, residence outside the corresponding metropolitan area. Women with current or recent pregnancy were rescheduled so that the first interview could take place ≥4 months after delivery.<br><br>In the SaMi-Trop cohort, exclusion criteria included pregnancy or breastfeeding, and any life-threatening disease with an ominous prognosis that suggested a life expectancy of <2 years.
Replication	The study consists of training and evaluating a Deep Neural Network for age prediction. The model was validated in a hold-out subsample not seen during training and two external cohorts.
Randomization	Randomization does not apply to the present study, due to its obsevational design.
Blinding	Blinding does not apply to the present study, due to its obsevational design.

Reporting for specific materials, systems and methods

We require information from authors about some types of materials, experimental systems and methods used in many studies. Here, indicate whether each material, system or method listed is relevant to your study. If you are not sure if a list item applies to your research, read the appropriate section before selecting a response.

Materials & experimental systems

n/a	Involved in the study	
☒	☐	Antibodies
☒	☐	Eukaryotic cell lines
☒	☐	Palaeontology and archaeology
☒	☐	Animals and other organisms
☐	☒	Human research participants
☒	☐	Clinical data
☒	☐	Dual use research of concern

Methods

n/a	Involved in the study	
☒	☐	ChIP-seq
☒	☐	Flow cytometry
☒	☐	MRI-based neuroimaging

Human research participants

Policy information about studies involving human research participants

Population characteristics	The CODE cohort consisted of mainly primary care patients over 16 years old that underwent a valid digital ECG of the TeleHealth Network of Minas Gerais, Brazil in 2010 to 2017. Minas Gerais had, in the census of 2010, a population of 19,597,330 persons. We included 1,558,415 patients of 811 counties of the state.<br><br>The ELSA-Brasil cohort included all active or retired employees of the six institutions (and, in a few instances, also of related educational or health institutions) from six Brazilian capitals, of both sexes, and with ages between 35 and 74 years.<br><br>SaMi-Trop is an NIH-funded prospective cohort of patients with chronic Chagas cardiomyopathy from 21 municipalities of the northern part of Minas Gerais State, Brazil. Eligible patients were selected based on the ECG results performed in 2011–2012 by the Telehealth Network of Minas Gerais. Only patients who fulfilled all of the following inclusion criteria were selected: (1) self-reported Chagas disease; (2) an index ECG reported as abnormal and (3) aged 19 years or more.<br>Table 1 summarizes the characteristics of the three cohorts analysed in this study.
Recruitment	In the CODE cohort, patients over 16 years old that underwent a valid digital ECG of the TeleHealth Network of Minas Gerais in 2010 to 2017 were included. Most of the remote points of the Telehealth Network of Minas Gerais are located in primary health care centers, although some ECGs machines are located in emergency units and ambulances. Clinical data were self reported and kept in the ECG database. No specific recruitment procedure was used.<br><br>In the ELSA-Brasil study, all active or retired employees of the six institutions (and, in a few instances, also of related educational or health institutions), of both sexes, and with ages between 35 and 74 years, were eligible for the study, totalling, in 2008, 52 137 potential participants. We chose civil servants as the source of the study population in order to minimize losses to follow-up related to geographical mobility. Volunteers were recruited through on-site and radio announcements, mailings, outdoor billboards and telephone calls, the latter more commonly used to attract retirees. A randomly ordered list of employees, stratified by sex, age and occupational category, was also used to actively recruit eligible participants. Efforts were made to recruit similar proportions of men and women as well as predefined proportions of age groups and occupational categories.<br><br>In the SaMi-Trop cohort, eligible patients were selected based on the ECG results performed in 2011–2012 by the Telehealth Network of Minas Gerais in 21 municipalities of the northern part of the state.
Ethics oversight	This study complies with all relevant ethical regulations. CODE Study was approved by the Research Ethics Committee of the Universidade Federal de Minas Gerais, protocol 49368496317.7.0000.5149. Since this is a secondary analysis of anonymized data stored in the TNMG, informed consent was not required by the Research Ethics Committee for the present study. ELSA-Brasil was approved by the Research Ethics Committees of the participating institutions and by the National Committee for Research Ethics (CONEP 976/2006) of the Ministry of Health. Sami-Trop study was approved by the Brazilian National Institutional Review Board (CONEP), No. 179.685/2012. In both investigations, all human subjects were adults who gave written informed consent. All researchers who deal with datasets signed terms of confidentiality and data utilization.

Note that full information on the approval of the study protocol must also be provided in the manuscript.
